# Ulinastatin alleviates early brain injury after intracerebral hemorrhage by inhibiting oxidative stress and neuroinflammation via ROS/MAPK/Nrf2 signaling pathway

**DOI:** 10.1590/acb370606

**Published:** 2022-09-05

**Authors:** Xi Wu, Wei Jiao, Junhui Chen, Yunna Tao, Jing Zhang, Yuhai Wang

**Affiliations:** 1 BS. 904th Hospital of Joint Logistic Support Force of PLA – Anhui Medical University – Wuxi Clinical College – Department of Neurosurgery – Wuxi, China.; 2 MD. 904th Hospital of Joint Logistic Support Force of PLA – Anhui Medical University – Wuxi Clinical College – Department of Neurosurgery – Wuxi, China.; 3 PhD. 904th Hospital of Joint Logistic Support Force of PLA – Anhui Medical University – Wuxi Clinical College – Department of Neurosurgery – Wuxi, China.

**Keywords:** Cerebral Hemorrhage, Brain Injuries, Oxidative Stress, Neuroinflammatory Diseases, Mice

## Abstract

**Purpose:**

Spontaneous intracerebral hemorrhage (ICH) is still a major public health problem, with high mortality and disability. Ulinastatin (UTI) was purified from human urine and has been reported to be anti-inflammatory, organ protective, and antioxidative stress. However, the neuroprotection of UTI in ICH has not been confirmed, and the potential mechanism is unclear. In the present study, we aimed to investigate the neuroprotection and potential molecular mechanisms of UTI in ICH-induced early brain injury in a C57BL/6 mouse model.

**Methods:**

The neurological score, brain water content, neuroinflammatory cytokine levels, oxidative stress levels, and neuronal damage were evaluated.

**Results:**

UTI treatment markedly increased the neurological score, alleviated brain edema, decreased the levels of the inflammatory cytokines tumor necrosis factor-α (TNF-α), interleukin-1β (IL-1β), IL-6, and NF-κB, decreased the levels of reactive oxygen species (ROS) and malondialdehyde (MDA), and upregulated the levels of glutathione (GSH), superoxide dismutase (SOD), and Nrf2. This finding indicated that UTI-mediated inhibition of neuroinflammation and oxidative stress alleviated neuronal damage after ICH. The neuroprotective capacity of UTI is partly dependent on the ROS/MAPK/Nrf2 signaling pathway.

**Conclusions:**

UTI improves neurological outcomes in mice and reduces neuronal death by protecting against neural neuroinflammation and oxidative stress.

## Introduction

Spontaneous intracerebral hemorrhage (ICH) is a common critical disease with high mortality and disability that accounts for 15-20% of all strokes, especially in elderly patients^1^-^4^. Large intracranial hematoma can lead to primary brain injury through the destruction of brain tissue and high intracranial pressure (ICP)^5^,^6^. In previous studies, craniotomies to remove hematomas were found to limit primary brain damage and decrease ICP after ICH^6^-^8^. However, hematoma evacuation shows no clinical benefit to patients and rarely affects neurological recovery^9^. Increasing evidence shows that red blood cell debris and its degradation products trigger secondary brain injury following ICH, and the mechanisms include neuroinflammation, oxidative stress, blood-brain barrier (BBB) damage, and neuron death^10^-^14^. According to previous studies^15^-^17^, treatment or drugs can improve neurological function after reducing mitochondrial apoptosis and decrease cerebral edema in mice after ICH.

Ulinastatin (UTI) is a serine protease inhibitor purified from human urine. UTI acts primarily against inflammation, regulates immunity, and protects organs^18^,^19^. He *et al*.^20^ reported that UTI plays an important role in pulmonary protective effects after cardiac surgery by meta-analysis. UTI can also alleviate cerebral ischemia-reperfusion injury and BBB permeability in animals^21^-^23^. However, no clear evidence has shown that UTI has an effect on early brain injury (EBI) during acute ICH, and its association with the levels of apoptotic molecules and oxidative stress remains to be elucidated.

In ICH, reactive oxygen species (ROS)-induced oxidative stress and glutamate-induced excitotoxicity can cause neuronal death rapidly in the brain^24^,^25^. The release of ROS after an extracellular or intracellular stimulus is crucial to the injury of neurons and tissues^6^. The transcription factor nuclear factor erythroid 2-related factor 2 (Nfe2L2, commonly referred to as Nrf2) regulates the expression of more than 250 genes and it is marked by its binding site, antioxidant response element (ARE)^26^. In acute central nervous system disease, Nrf2 can also modulate the cellular antioxidant response and mitigate electrophilic or oxidative stress, inflammation, proteostasis, xenobiotic/drug metabolism, iron/heme metabolism, carbohydrate, and lipid metabolism^6^. Due to cerebral tissue’s higher oxygen consumption than most other organs, excessive ROS are generated after hemorrhage as a result of oxidative stress^27^. It would be valuable to investigate new potential drug goals by targeting the ROS/MAPK/Nrf2 signaling pathway in oxidative stress and neuroinflammation.

In the present study, we constructed a mouse ICH model to study the effects of UTI on EBI and explored the crosstalk between oxidative stress and neuroinflammation. We also explored the mechanism by which the ROS/MAPK/Nrf2 signaling pathway may regulate this process.

## Methods

All animal experiments performed in this study complied with the National Institutes of Health guidelines for the handling of laboratory animals and were approved by the Ethics Committee of the Wuxi Medical College of Anhui Medical University (YXLL-2021-A15). A total of 60 healthy adult male C57BL/6J mice (n = 20/group; age 8-10 weeks old; Anhui Medical University, Hefei, China) weighing between 22-25 g were used when conducting all the experiments for the current study. The mice were housed in animal care facilities on a 12-h light/dark cycle and had free access to food and water.

### Animal intracerebral hemorrhage model

The ICH mouse model was generated based on a previously described protocol involving autologous blood injection^28^. Briefly, male C57BL6/J mice were anesthetized by intraperitoneal (i.p.) injection of 50 mg/kg pentobarbital sodium and placed in a prone position with a stereotactic head frame. The rectal temperature was kept at 37 ± 0.5°C during the operation using a heating pad. An artificial tear ointment was used to protect the eye from injury during surgery. A midline scalp incision was made, and a cranial burr hole with a 1-mm diameter was made at the following coordinates relative to bregma: 0.2 mm posterior, 2.2 mm lateral to bregma, and 3.5 mm below the dura. A total of 30 μL of autologous blood without anticoagulation was collected from the caudal artery and rapidly injected into the basal ganglia through the burr hole via the 26-gauge needle of a 10-μL Hamilton syringe. First, 5 μL of arterial blood was injected at a depth of 2.8 mm from the dura (injection speed: 3 μL/min). Five minutes later, the other 25 μL of blood was injected at a depth of 3.5 mm (injection speed: 3 μL/min). After the injection of autologous blood, the needle was kept in the brain for 10 min to prevent blood backflow along the needle tract. Finally, the hole was covered with medical bone wax. The animals in the sham group received similar surgical procedures, but they were injected at the same site with an equal volume of sterile saline instead of blood.

### Drug administration

UTI (Techpool Biochem, Guangdong, China) was stored at 4°C and dissolved in 0.9% normal saline when used. UTI (10^4^ U/kg) was administered by intraperitoneal injection before the onset of ICH^29^.

### Neurobehavioral assessment

Neurobehavioral assessment at 72 hours after ICH was performed according to a previous study^30^. The neurological scores were summed to calculate the neurological scores, which ranged from 0 to 18 points. Neurological dysfunction was represented by a higher score. An independent observer was blinded in this study.

### Brain water content measurement

Brain water content measurement was detected using the standard wet-dry method, according to previous studies^31^-^33^. After mice were sacrificed, the entire brain was harvested and separated into different parts, and the wet weight was acquired by precision balance and then dehydrated at 105°C for 24 hours to acquire the dry weight (Eq. 1).


Rain water content(%)=(Wet weight−Dry weight)/Wet weight×100% (1)

### Evans blue extravasation

The method of Evans blue extravasation was performed according to a previous study^34^. Briefly, mice were anesthetized 72 hours after ICH. Evans blue dye (2%, 5 mL/kg; Sigma–Aldrich, St. Louis, MO, United States of America) was injected into the left femoral vein and circulated for 60 min. Then, phosphate-buffered saline (PBS) was intracardially perfused after the mice were sacrificed. The brains were removed, weighed, homogenized in saline, and centrifuged at 15,000 × g for 30 min. Subsequently, the resultant supernatant was added to an equal volume of trichloroacetic acid, incubated overnight at 4°C, and centrifuged at 15,000 × g for 30 min. Next, the resultant supernatant was collected and spectrophotometrically quantified at 610 nm for Evans blue dye.

### Analysis of reactive oxygen species

The nonfluorescent diacetylated 2',7'-dichlorofluorescein (DCF-DA) probe (Sigma–Aldrich), which becomes highly fluorescent upon oxidation, was used to evaluate intracellular ROS production according to the manufacturer’s instructions^35^.

### Analysis of lipid peroxidation

Malondialdehyde (MDA), GSH, and superoxide dismutase (SOD) levels were detected with a corresponding assay kit (Nanjing Jiancheng Bioengineering Institute) according to the manufacturer’s instructions^36^.

### Cytokine measurements of ipsilateral cortex tissue

The levels of interleukin-1β (IL-1β) (cat. no. ab197742; Abcam), IL-6 (cat. no. ab222503; Abcam), tumor necrosis factor-α (TNF-α) (cat. no. ab208348; Abcam), and NF-κB (cat. no. ab176663; Abcam) were measured by enzyme-linked immunoassay (ELISA) according to the manufacturer’s instructions^37^.

### TUNEL staining

TUNEL staining was used to evaluate neuronal death in the hippocampus according to a previous study^32^. Brain specimens were immersed in a TUNEL mixture for 2 h at 37.5°C, followed by staining with DAPI. The procedure was performed with a TUNEL staining kit according to the manufacturer’s instructions.

### Western blot analysis

Western blot analyses were performed as previously described^31^. Briefly, cerebral cortex samples were collected, homogenized, and extracted in RIPA lysis buffer containing a 1% (v/v) protease and phosphatase inhibitor cocktail. The protein samples were separated by SDS–PAGE and transferred to PVDF. After blocking with 5% (w/v) nonfat milk, the membranes were incubated with the following primary antibodies overnight at 4°C: rabbit anti-β-actin (1:1,000, rabbit polyclonal, Abcam, ab8227), rabbit ZO-1 (1:1,000, rabbit polyclonal, Abcam, ab 96587), phospho-p38 (CST, #4551, 1:2,000), and rabbit anti-Nrf2 (1:1,000, rabbit polyclonal, Abcam, ab92946). Afterwards, the membranes were incubated with HRP-conjugated goat anti-rabbit IgG or goat anti-mouse IgG secondary antibodies (1:5,000) for 1 h at room temperature and then scanned with a Bio-Rad (California, United States of America) gel imaging system.

### Quantitative real-time polymerase chain reaction

Quantitative real-time (qRT) polymerase chain reaction (PCR) analysis was performed according to a previous study^38^. Total RNA was extracted using TRIzol Reagent (Gibco; Thermo Fisher Scientific, Inc., Waltham, MA, United States of America) according to the manufacturer’s instructions. Then, a miScript cDNA synthesis kit (K1622; Thermo Fisher Scientific Inc., Rockford, IL, United States of America) was used for the reverse transcription reaction. For qRT-PCR, a 7,500 real-time PCR thermocycler (Applied Biosystem) was used. Real-time RT-PCR was performed in a total volume of 10 μL containing 1 μL of cDNA, 0.6 μL of primers, and 8.4 μL of SYBR Green Master Mix (Toyobo Co., Ltd., Osaka, Japan). The program steps were 30 s at 95°C, 40 cycles of 5 s at 95°C, and 30 s at 60°C, followed by melt curve analysis. Gene expression was quantified with standard samples and normalized to GAPDH. The target genes and the specific primers are as follows:

- Nrf2 (forward, 5’- ATCACGAGCCCTGAAACCAA-3’; reverse, 5’- GGCTGCAAAATGCTGGAAAA-3’);
- MAPK (forward, 5’- TGTGTTCACCCCTGCCAAGT-3’; reverse, 5’- GCCCCCGAAGAATCTGGTAT-3’);
- GAPDH (forward, 5’- ATGGGTGTGAACCACGAGA-3’ and reverse, 5’- CAGGGATGATGTTCTGGGCA-3’).

### Statistical analysis

All data are presented as the means ± standard deviations. All statistical analyses were performed using GraphPad Prism 6 (GraphPad Software, Inc., San Diego, CA, United States of America). After checking for normal distribution, differences between two groups were analyzed with Student’s t-test (two-tailed), and data were analyzed by one-way analysis of variance (ANOVA) with post hoc Tukey’s test or Dunnett’s test applied to assess multiple comparisons. Nonparametric data were analyzed using Kruskal-Wallis H analysis followed by a Mann-Whitney U test, and p < 0.05 was considered statistically significant.

## Results

### Ulinastatin alleviates early brain injury after intracerebral hemorrhage

**Figure 1:**
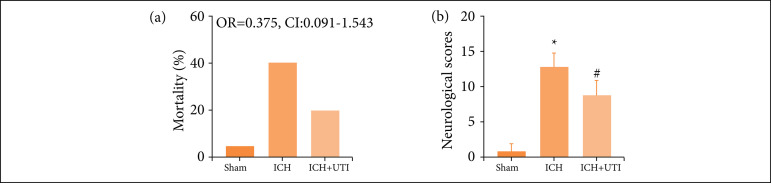
UTI alleviates neurological deficits and mortality after ICH. **(a)** Comparison of the mortality between the three groups. **(b)** Neurological scores of mice in the sham group, ICH group, and ICH group treatedwith UTI at 72 h (n = 10, analysis of variance; means ± standard error of mean).

ICH: intracerebral hemorrhage; UTI: Ulinastatin; OR: odds ratio; CI: confidence interval; *P < 0.05 *vs*. sham; ^#^P < 0.05 *vs*. ICH.

The results showed that ICH significantly increased the mortality rates (Fig. 1a). This effect was alleviated by UTI treatment, while there was no significant difference among the three groups. We also found that neurological scores increased significantly after ICH, and UTI administration significantly improved neurological function (Fig. 1b).

### Ulinastatin alleviates brain edema and blood-brain barrier permeability after intracerebral hemorrhage

**Figure 2:**
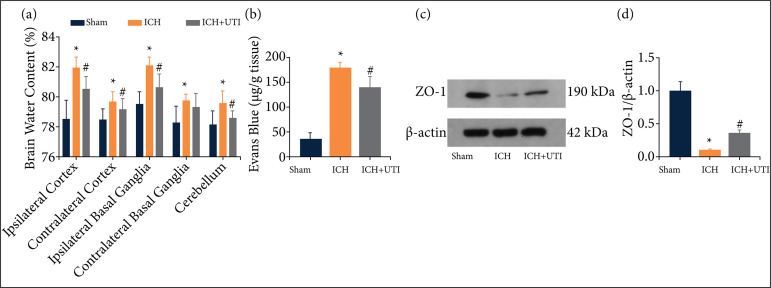
UTI alleviates brain edema and BBB permeability after ICH. **(a)** UTI alleviates brain water content after ICH.**(b)** UTI alleviates BBB permeability after ICH. **(c)** Expression of ZO-1 was determined by Western blotting. **(d)** Quantification of ZO-1 to β-actin loading control (n = 5; analysis of variance; means ± standard error of mean).

ICH: intracerebral hemorrhage; UTI: Ulinastatin; BBB: blood-brain barrier; *P < 0.05 *vs*. sham; ^#^P < 0.05 *vs*. ICH.

The results showed that ICH significantly increased the brain water content, which was alleviated after UTI treatment (Fig. 2a). BBB permeability was increased significantly after ICH, and UTI administration significantly alleviated this effect (Fig. 2b). The expression levels of Zonula occludens-1 (ZO-1) were detected by Western blotting (Fig. 2c). The results showed that ZO-1 (Fig. 2d) was downregulated significantly after ICH, while UTI treatment markedly alleviated this decrease.

### Ulinastatin alleviates neuronal apoptosis after intracerebral hemorrhage

**Figure 3:**
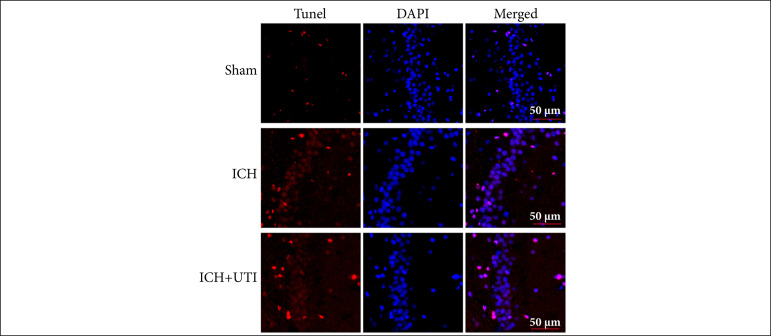
UTI alleviates neuronal apoptosis after ICH. TUNEL staining showed that UTI alleviated neuronal apoptosis in the hippocampus at 72 h after ICH, and representative images of apoptotic neurons are shown. Scale bar = 50 μm.

ICH: intracerebral hemorrhage; UTI: Ulinastatin.

The TUNEL assay results showed more hippocampal neuronal damage after ICH, which was reversed after UTI administration (Fig. 3). Based on these results, UTI can alleviate hippocampal neuronal damage after ICH.

### Ulinastatin alleviates neuroinflammation after intracerebral hemorrhage

**Figure 4:**
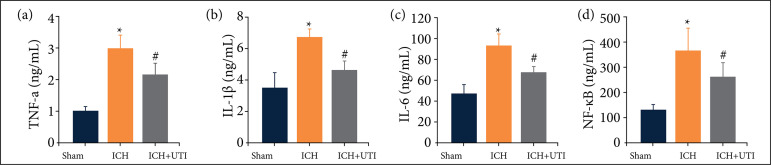
UTI alleviates neuroinflammation after ICH. UTI significantly reduced hippocampal **(a)** TNF-α, **(b)** interleukin-1β (IL-1β), **(c)** IL-6, and **(d)** NF-κB levels at 72 h after ICH (n = 5, analysis of variance; means ± standard error of mean).

ICH: intracerebral hemorrhage; UTI: Ulinastatin; TNF-α: tumor necrosis factor-α; IL: interleukin; *P<0.05 vs. sham; ^#^P<0.05 vs. ICH

As previous studies have identified a vital role for neuroinflammation in EBI after ICH, increased neuroinflammation aggravates EBI. The inflammatory complex induces the secretion of proinflammatory cytokines, including IL-1β, IL-6, and TNF-α, and the subsequent activation of pro-inflammatory signaling through NF-κB initiates pyroptosis. Therefore, we measured the hippocampal levels of IL-1β, IL-6, TNF-α, and NF-κB using ELISA. The levels of proinflammatory cytokines increased significantly after ICH, while the levels of proinflammatory cytokines decreased significantly after UTI treatment (Figs. 4a-4d). Hence, these results suggested that UTI exhibited potent anti-inflammatory activity against ICH-induced neuroinflammation.

### Ulinastatin inhibits intracerebral hemorrhage-induced oxidative stress in the hippocampus

**Figure 5:**
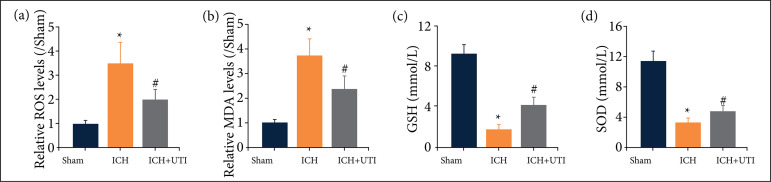
UTI inhibits ICH-induced oxidative stress in the hippocampus. **(a)** The levels of ROS by the DCF-DA method. (**b-d**) The levels of MDA, GSH, and SOD were quantified by using commercial kits (n = 5, mean ± standard error of mean).

ICH: intracerebral hemorrhage; UTI: Ulinastatin; ROS: reactive oxygen species; MDA: malondialdehyde; GSH: glutathione; SOD: superoxide dismutase; *P<0.05 vs. sham; ^#^P<0.05 vs. ICH

To understand the neuroprotective mechanism of UTI, we focused on its antioxidation effect. We detected the levels of ROS, GSH, SOD, and MDA (Figs. 5a-5d). The levels of both ROS and MDA increased after ICH, but they decreased significantly after UTI treatment. The levels of GSH and SOD decreased after ICH, but they increased significantly after UTI treatment. Hence, these data showed that UTI can inhibit oxidative stress activation after ICH.

### Ulinastatin regulates oxidative stress and neuroinflammation via ROS/MAPK/Nrf2 signaling pathway

**Figure 6:**
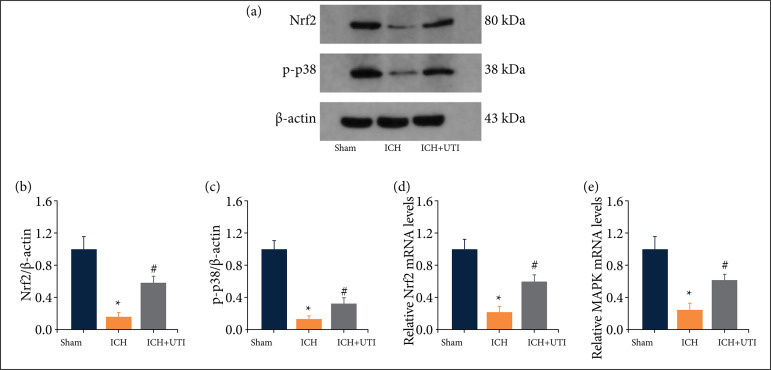
UTI regulates oxidative stress and neuroinflammation by modulating the ROS/MAPK/Nrf2 signaling pathway after ICH.**(a)** Levels of Nrf2 and MAPK after ICH were determined using Western blotting. **(b)** Quantification of Nrf2 levels to β-actin, the loading control. **(c)** Quantification of MAPK (p-p38) levels to β-actin. **(d)** Levels of Nrf2 mRNA were measured by real-timePCR. **(e)** Levels of TMAPK mRNA were measured by real-time PCR (n=5, mean ± standard error of mean).

ICH: intracerebral hemorrhage; UTI: Ulinastatin; *P < 0.05 *vs*. sham; ^#^P < 0.05 *vs*. ICH; PCR: polymerase chain reaction.

ROS/MAPK/Nrf2 is a core signaling pathway of oxidative stress and neuroinflammation. According to previous studies^39^,^40^, activation of MAPK/Nrf2 signaling pathway is partially dependent on ROS production. We detected the levels of MAPK and Nrf2 proteins by performing Western blotting (Fig. 6a). The levels of MAPK and Nrf2 decreased significantly in the ICH group and increased after UTI administration (Figs. 6b-6c). Additionally, real-time PCR also demonstrated a similar result (Figs. 6d-6e). Thus, these results showed that UTI may exert neuroprotection by regulating the MAPK/Nrf2 signaling pathway.

## Discussion

**Figure 7:**
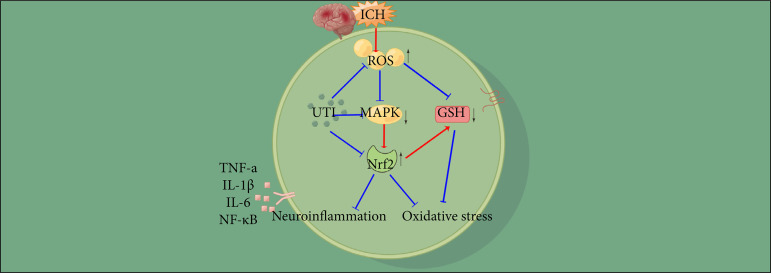
A diagram of the proposed model explaining the observations of ROS/MAPK/Nrf2-mediated regulation of oxidative stress and neuroinflammation after ICH and potential mechanisms underlying the effect of UTI intervention.

ICH: intracerebral hemorrhage; ROS: reactive oxygen species; UTI: Ulinastatin; GSH: glutathione; TNF-α: tumor necrosis factor-α; IL: interleukin.

Here, we evaluated the therapeutic potential of UTI to alleviate EBI in a mouse model of ICH. As shown in the present study, UTI is a neuroprotective agent that attenuates early brain injury following ICH. We found that UTI improves neurological dysfunction after ICH, alleviates brain damage in a mouse ICH model, relieves neuroinflammation after ICH and then decreases inflammatory damage in the brain, prevents oxidative stress after ICH and alleviates neuronal death, and the antiapoptotic and antioxidative stress effects of UTI may be related to the ROS/MAPK/Nrf2 signaling pathway (Fig. 7).

UTI is a 67 kDa glycoprotein purified from the urine of healthy humans that is a nonspecific protease inhibitor and a urinary trypsin inhibitor used to treat acute inflammatory disorders, sepsis, toxic shock, and hemorrhagic shock^41^,^42^. Recent studies have demonstrated that UTI can alleviate cerebral ischemia-reperfusion injury by regulating inflammation and oxidative stress^21^,^43^. However, the neuroprotective effect of UTI in ICH is unclear and lacks related clinical studies. Liu *et al*.^19^ reported that UTI can decrease the brain water content and BBB permeability significantly after ICH, possibly through7 decreased activation of astrocytes and ET-1, inhibiting the expression of proinflammatory VEGF and MMP-9. Another study also reported that UTI can attenuate brain edema after ICH in male Sprague-Dawley rats. The preliminary molecular mechanism may be through a decrease in the expression level of aquaporin-4 (AQP4) and proinflammatory cytokines, including IL-1β and TNF-α, as well as the activity of NF-κB^44^. In the present study, we also demonstrated that UTI can alleviate brain edema, improve neurological function, relieve the neuroinflammatory response, and decrease hippocampal neuronal damage and oxidative stress.

The mechanism of neuroprotection of UTI is multiple and complicated^18^,^19^. Cui *et al*.^21^ reported that UTI regulates inflammation and oxidative stress in ischemia-reperfusion (I/R) injury, possibly through the Nrf-2/HO-1 pathway. Li *et al*.^22^ also demonstrated that UTI can alleviate ischemic injury by improving BBB permeability by decreasing the expression of MMP-9 and increasing ZO-1. Koga *et al*.^45^ showed that UTI can inhibit ROS generation, prevent oxidative stress, and alleviate early inflammation in an ischemia/reperfusion model. Additionally, UTI can also improve hippocampal endoplasmic reticulum stress and apoptosis^46^. In central nervous system disease, the neuroprotection of UTI is partly dependent on the inhibition of apoptosis, neuroinflammation, and oxidative stress^43^,^47^,^48^.

The molecular mechanisms of oxidative stress and neuroinflammation are complex. The present study showed that UTI decreases the levels of ROS production, subsequently upregulating the expression levels of MAPK and Nrf2 and alleviating the activation of oxidative stress and neuroinflammation. ROS play a vital role in neuronal or other tissue injury and are generated by extracellular or intracellular stimuli after oxidative stress^6^. UTI can increase the nuclear translocation of Nrf2, stimulate Nrf2 DNA binding activity, and then upregulate the expression levels of HO-1, decrease airway inflammation, alleviate tissue injury, reduce oxidative stress, and enhance antioxidant enzyme activities in allergic inflammatory diseases^49^. Li *et al*.^50^ reported that UTI pretreatment can significantly suppress LPS-induced ROS production by activating the nuclear translocation of Nrf2 via promotion of p62-associated Keap1 degradation and then inhibiting inflammation and oxidation. In a cerebral ischemia model, UTI enhances Nrf2-ARE signals and inhibits products of oxidative stress in the hippocampus, which can improve neurological deficiencies^51^.

Additionally, activated p38 MAPK could facilitate the disassociation of Nrf2 from Keap1 to initiate the transcription of several anti-apoptotic, anti-ferroptosis, and antioxidant genes^6^,^52^. Li *et al*.^53^ demonstrated that UTI can alleviate brain injury by regulating the expression of TLR4 and NF-κB. Cui e Zhu^44^ also concluded that UTI therapy in traumatic brain injury decreased the activities of IL-1, TNF-, and NF-B.

In the present study, we observed that UTI can alleviate EBI after ICH by regulating crosstalk between oxidative stress and neuroinflammation by mediating ROS generation and via the MAPK/Nrf2 signaling pathway. The specific mechanism remains unclear. Additionally, this experiment was conducted in mice, and the effectiveness of the treatment in humans remains debated. Further exploration of the clinical effects of UTI on ICH patients will be conducted in the future.

## Conclusions

Oxidative stress and neuroinflammation are mediated by ROS and play an important role in EBI after ICH. UTI-mediated regulation of oxidative stress and neuroinflammation is partly dependent on the ROS/MAPK/Nrf2 pathway and provided a new idea to explore the biological effects and mechanisms underlying the antioxidative stress, anti-inflammatory, and neuroprotective properties of UTI.
